# Influence of solution pH dependency on structure, optical with photoelectrochemical characteristics of SILAR deposited copper oxide thin films

**DOI:** 10.1016/j.heliyon.2024.e33579

**Published:** 2024-06-25

**Authors:** E. Arulkumar, S. Thanikaikarasan, S. Rajkumar, Wasihun Wondimu

**Affiliations:** a Department of Physics, Saveetha School of Engineering, Saveetha Institute of Medical and Technical Sciences, Chennai, 602 105, Tamil Nadu, India; b Department of Mechanical Engineering, Faculty of Manufacturing, Institute of Technology, Hawassa University, Hawassa, Ethiopia

**Keywords:** CuO, EIS, Linear sweep voltammetry, Photoelectrochemistry, SILAR, Thin films

## Abstract

Photoelectrochemical (PEC) technology is a promising approach for converting solar energy into chemical energy, offering significant potential for renewable energy applications. In this work, the CuO thin film was fabricated with different pH value in between 8.5 ± 0.1 and 10.5 ± 0.1 via Successive Ionic Layer Adsorption and Reaction (SILAR) method. The Effect of pH on thickness, structural, morphological, elemental composition and optical properties were investigated by using stylus profilometry, XRD, SEM, TEM, EDX, UV–vis and PL. The XRD results showed that as the pH increased, the crystallite size increased from 19.24 nm to 25.62 nm, with a monoclinic phase along the (111) direction. The CuO film deposited at pH value 10.5 ± 0.1 exhibit well defined identical particle with its size in the range between 200 and 300 nm was confirmed by SEM and TEM analysis. As the pH increased from 8.5 ± 0.1 to 10.5 ± 0.1, the CuO film bandgap (E_g_) value reduced from 1.52 eV to 1.42 eV with indirect transition. The CuO photocathode deposited at pH 10.5 ± 0.1 shows maximum photocurrent density of 1.45 mA/cm^2^ at −0.1 V vs. RHE in 0.5 M Na_2_SO_4_ solution. Furthermore, the Electrochemical Impedance Spectroscopy (EIS) analysis shows, the CuO (pH 10.5 ± 0.1) electrode have higher conductivity value of 0.6862 S/cm compared CuO at pH 8.5 ± 0.1 (0.2779 S/cm) and CuO at pH 9.5 ± 0.1 (0.4646 S/cm) electrodes.

## 1 Introduction

The growing global demand for energy is being largely met by the consumption of fossil fuels. This dependence on petroleum product, coal, oil and natural gas is causing a number of negative impacts on the environment [1]. It is critical to identify environmentally feasible alternative sources of green energy. The process of solar water splitting is indeed a highly promising approach for producing hydrogen as a renewable energy source. This process involves using sunlight to split water molecules (H_2_O) into hydrogen (H_2_) and oxygen (O_2_) through a photoelectrochemical (PEC) cell or a photovoltaic-electrolysis system [2]. A wide range of materials have been extensively investigated for possible use as photoanodes and photocathodes in the form of thin layers and nanostructures. In this perspective, semiconductor contributed PEC water splitting might be among the most feasible ways for addressing the world energy need since it can create H_2_ via the cathode [3]. Indeed, employing semiconductor-assisted PEC water splitting has the potential to contribute significantly to reducing CO_2_ emissions and greenhouse gas levels [4]. This method provides a more environmentally friendly alternative by generating H_2_ as a clean energy source, thereby contributing to the prevention of climate change impacts. In 1972, Honda and Fujishima performed ground breaking study in the domain PEC water splitting employing TiO_2_ as a photoanode [5]. Following this, enormous efforts have been spent to building efficient semiconductor photoelectrodes for better solar to hydrogen efficiency up to now. The actual use of semiconductor nanomaterials is still hindered by certain issues. Presently, there is substantial research focused on identifying suitable materials for constructing stable photocathodes with optimal band gaps and excellent semiconductor properties. Lee et al. [6] revealed hematite, tungsten oxide, bismuth vanadate, titanium oxide, and copper oxides, based photoanodes and photocathodes in PEC cells. The p-type copper-based and iron-based metal oxide photocathodes show promise, they also face challenges related to stability and charge separation reported by Yang et al. [7]. Pessoa et al. [8] reviewed TiO_2_ and/or SiC in PEC applications. He et al. [9] indicated ternary metal oxide semiconductors as potential photoelectrode. Trunk et al. [10] reported ZnO-based photoanodes for PEC applications. Kumar et al. [11] and Bellani et al. [12] both reported copper and carbon-based materials, as well as ways of addressing efficiency and operational challenges, including the use of protective and charge-selective layers. Further Zhang et al. [13] and Deng et al. [14] reported Cu_2_O-based construction of multi-materials and metal-organic framework coatings. Wang et al. [15] emphasizes stability in photocathode design, energy band alignment and protective layers. For semiconductor nanoparticles to be able to capture a significant amount of direct sunlight light, they need to have specific characteristics, like an appropriate band gap. Furthermore, these materials must be stable under sustained light conditions [16,17]. For implementation as photoanodes in PEC water splitting applications, a variety of stable and effective metal oxide semiconductors, such as TiO_2_ [18], ZnO [19], WO_3_ [20], Fe_2_O_3_ [21] and BiVO_4_ [22]. Indeed, several copper oxides based photocathodes have studied such as CuO [23], Cu_2_O [24], CuWO_4_ [25], CuFe_2_O_4_ [26], CuNb_3_O_8_ [27] and CuO–Cu_2_O [28] etc. The Cu_2_O is a non-stoichiometric semiconductor that is abundant and exhibits a direct band gap of approximately 2 eV with p-type conductivity and high absorption coefficient make it of interest for applications in solar energy conversion and PEC devices [29,30]. One of the challenges associated with using Cu_2_O and CuO films as photocathodes in PEC cells is their susceptibility to photocorrosion in the presence of an electrolyte. The redox potential levels for self-reduction and self-oxidation reactions are situated within the band gap, it implies that these processes can occur under the influence of light, contributing to the transformation of Cu_2_O into Cu or CuO [3,31]. Such reactions can lead to changes in the materials structure and composition, potentially impacting its performance as a photocathode in PEC applications. The limitations posed by these energetic processes emphasize the importance of addressing the stability issues to ensure the long-term functionality of Cu_2_O in PEC applications for hydrogen generation [32,33]. The use of barrier layers or protective coatings is a common strategy in materials science to address issues related to stability, corrosion, and degradation [34]. By implementing such protective techniques, researchers aim to extend the operational life and efficiency of Cu_2_O-based photocathodes, making them more viable for sustainable energy applications. Certainly, CuO is another semiconductor material with promising characteristics that could be used in sunlight-driven water splitting systems. CuO have an optical band gap (Eg) of approximately 1.5 eV, a higher coefficient of absorption and exhibits p-type conductivity [35]. Additionally, the compounds will be crystalline considering the direct band gap is greater than the indirect band gap in accordance, the direct band gap value of CuO is between 3.50 and 3.85 eV, while the indirect band gap value is between 1.2 and 1.4 eV [[36], [37], [38]]. Aditionally, CuO is an important compound of the metal oxide semiconductors which contains both XI and XVI group elements which exhibits three stoichiometric - nonstoichiometric crystalline phases like monoclinic [39], cubic [[40], [41], [42]] and hexagonal [43]. These features make it suitable for use as a photoanode in PEC cells aimed at solar water splitting for hydrogen generation. To improve the stability and efficiency of CuO as a photoanode, researchers have employed various strategies. One approach involves adding Au–Pd nanostructures as co-catalysts. These nanostructures act as promoters, facilitating the water-splitting reaction and improving the overall efficiency of the photoanode [44]. Additionally, modifying the stoichiometry of CuO by introducing an excess of oxygen has been explored [45]. This modification aims to optimize the electronic structure and surface properties of CuO, contributing to increased stability and improved performance in the challenging environment of solar water splitting. Further research has been directed towards the production of heterostructures based on the combination of CuO and Cu_2_O [46]. Heterostructures involve the integration of two or more different semiconductor materials with distinct properties. In the case of CuO/Cu_2_O heterostructures, this combination leverages the individual characteristics of both materials to enhance the overall performance in PEC applications. The fabrication of copper oxide based thin films by various technique such as magnetron sputtering [47], chemical vapour deposition [48], spin coating [49], electrodeposition [50] and SILAR [51]. All the features mentioned above make copper oxides an efficient visible light active photocathode for PEC water splitting applications. These reports entail a cost-intensive process for producing copper oxide thin films. Among other techniques, the SILAR approach has several advantages, including low-cost, simplicity, large-scale deposition, generating feature layer by layer, convenience of use, and repeatability. Furthermore, the film thickness is controlled by a deposition process like pH, temperature, and dipping-drying duration [52]. The deposition process, size distribution, and distinctive precursor of anionic and cationic solutions all contributed to the efficiency of material use. These films have been employed in a variety of possible application fields, including solar cells, gas sensors, energy storage, transistors, diodes, and water splitting systems. Several research reports showed that how different methodologies found to enhance the CuO film formation and properties [53,54]. As far as we are aware that how experimental factors affects CuO thin film deposition using the SILAR method. The pH adjustment tends to control the formation of particles. Lower pH values cause insufficient particle formation, whereas higher pH values reduce unreacted ions. This suggests that optimal particle formation occurs within a specific pH range. This study investigated how pH levels influence the crystallinity, morphology and optical properties of CuO films. Additionally, their electrical properties and PEC performance are investigated.

## 2 Experimental

### 2.1 Materials used

Cupric acetate monohydrate (Cu (CH_3_COO)_2_. H_2_O), purchased from Loba Chemie (AR Grade), was used as a precursor. Ammonium hydroxide (NH_4_OH extra pure AR, 30 %) was used to adjust the pH. Deionized water was used as a solvent. Chromic acid, acetone and ethanol were used for substrate cleaning purposes. Indium-doped tin oxide (ITO) substrates with a sheet resistance of 20 Ω/sq and a thickness of 1.1 mm were also used.

### 2.2 Preparation of CuO thin films

In SILAR method, CuO thin film was prepared using different copper precursors solution. Gençyılmaz et al. [39] reported 0.3 M of [CuCl_2_.2H_2_O] in 200 mL of water was used to form [Cu (NH_3_)_4_]^2+^. Furthermore, the oxidizing liquid containing 200 mL of deionized water kept at 90 °C was used and the rinse carried out using the deionized water. Daoudi et al. [55] reported Holmarc's SILAR coating system using 0.1 M of (CuCl_2_·2H_2_ O) to form copper-ammonia complex for deposition of CuO thin film. Shinde et al. [56] reported four beaker SILAR system with 25 mL of 0.05 M of CuSO_4_ 2H_2_O as cationic and 25 mL of 0.02 M of NaOH as anionic solution were used for to form CuO thin film. Rajini et al. [57] reported comparison of conventional SILAR and modified SILAR method. The reports mentioned above have employed different factors such as bath temperature, dipping-drying time, pH levels, and the use of both cationic and anionic solutions. According to these reports, in the conventional SILAR technique, anionic and cationic solutions are typically kept in separate beakers. A modified SILAR technique involves combining all of the precursor solutions in a single beaker. This modification aims to simplify the process, reducing both the workload and time required for the deposition process. In this work, the modified SILAR method was used to prepare CuO thin films on ITO substrate. There are two beaker systems involved for in this technique. The detailed experimental procedure as following, the cationic solution was made by dissolving 2.45 g (0.25 M) of Cu (CH_3_ COO)_2_. H_2_O in 50 mL distilled water. After just a few minutes of atmospheric stirring, the solution's pH was adjusted to 8.5 ± 0.1 by adding 0.1 N of NH_3_OH, which produced the copper-ammonia complex [Cu(NH_3_)_4_ (H_2_O)_2_]^2+^ needed for the formation of CuO thin films.

**Fig. 1:**
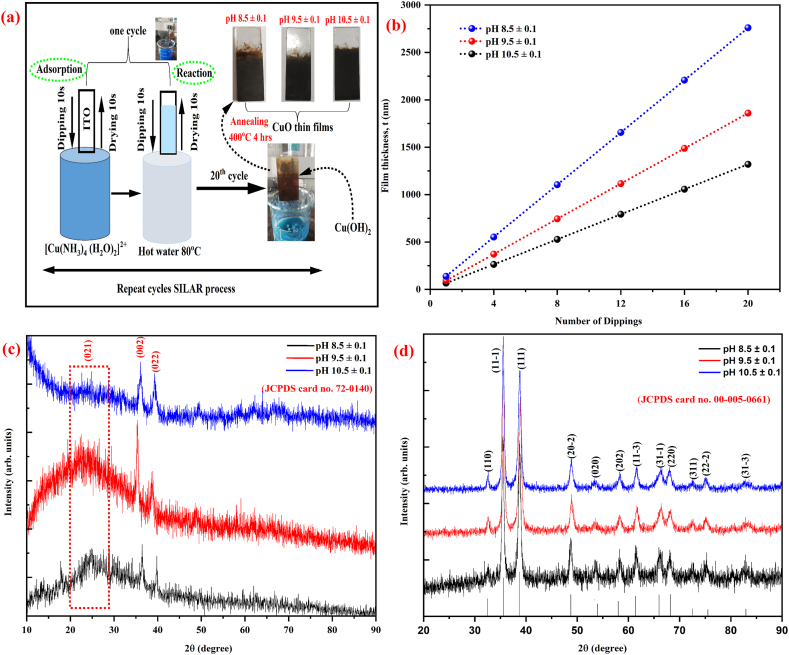
(a) Experimental procedure for deposited CuO thin film, (b) Film thickness with number of dippings, (c) XRD pattern of non-annealed thin film and (d) XRD pattern of annealed CuO thin film at different pH values 8.5 ± 0.1, 9.5 ± 0.1 and 10.5 ± 0.1.

The anionic solution was maintained at 80 °C. Before utilization, ITO surface underwent a thorough cleaning process involving a 15-min ultrasonic treatment in hot chromic acid, acetone and ethanol. Subsequently, they were immersed in distilled water and air-dried before being deployed for deposition. Microspores form on the substrate's surface as an outcome of this process. The cleaned ITO substrates were immersed into [Cu(NH_3_)_4_ (H_2_O)_2_]^2+^ for 10 s, then dried for 10 s, then again immersed in hot distilled water (80 °C) for another 10 s, and finally dried in air for 10 s before the start of the next deposition cycle, this completes one deposition cycle. This same process was continuously repeated 20 times, to form Cu (OH)_2_ particles on the surface of the substrate. Fig. 1(a) shows the schematic representation of deposition of CuO thin films by modified SILAR technique. Here, the pH of the solution plays an important role for the deposition process. According to the SILAR method, no particle form on the substrate surface when the solution pH is less than 7 (acidic medium). But the pH of the solution increases above 7 (alkali medium) precursor solution may begin to form particles with a gel-appearance. The formed particle can now begin to precipitate. Hence, the stirring process prevented the particle precipitation. Following a series of dipping and drying steps, a thin layer is created on the microspore surface of the ITO substrate. Here, the particle has already formed with some shape, while the pH adjustment. Through the process of dipping and drying, the formed particle was adhered on the surface of the ITO substrate with layer by layer. In accordance, our experiment the cleaned ITO substrate was rinsed in the prepared solution for 10 s at ambient temperature leads to produce Cu (OH)_2_ particles those are adsorbed on the surface of ITO. Subsequently the substrate was rinsed by deionized water at 80 °C for 10 s to remove the loosely bounded Cu (OH)_2_ particles and to get well adherent nature on surface of the substrate. The same process was used to prepare CuO thin film with different pH value (pH = 8.5 ± 0.1, 9.5 ± 0.1 and 10.5 ± 0.1). The prepared all Cu (OH)_2_ films was annealed at 400 °C for 4 h to form CuO and the basic reactions for formation the CuO thin films as following Eqs. (1), (2), (3), (4)

As shown in Eq. (1), represents the formation of copper ammonia complex

(1) $${\left[Cu{\left(NH_{3}\right)}_{4}{\left(H_{2}O\right)}_{2}\right]}^{2+}+4H_{2}O\to Cu^{2+}+4NH_{3}+4OH^{-}$$

Formation of copper hydroxide is given in Eq. (2)

(2) $$Cu^{2+}+2OH^{-}\to Cu{\left(OH\right)}_{2}$$

The equilibrium relationship between copper hydroxide and copper oxide is shown in Eq. (3)

(3) $$Cu{\left(OH\right)}_{2}\Leftrightarrow {\left[Cu^{2+}+\left(2O^{2-}+H^{+}\right)\right]}_{solid}$$

From Eq. (4), the oxidation of copper hydroxide to copper oxide formation represented

(4) $${\left[Cu^{2+}+O^{2-}\right]}_{solid}+\left(O^{2-}+2H^{+}\right)\overset{400^{o}C}{\to }CuO_{solid}+H_{2}O$$

### 2.3 Characterization

The deposited films thickness was measured by using SJ-301 Mitutoyo Surface Profilometer (Needle angle by 45° with scanning speed 0.3 mm/min, Stylus tip radius 2 μm, Evolution length 1.25 mm and error not exceed ± 20 nm). The crystallinity was analysed BRUKER USA D8 Advance, Davinc X-ray diffractometer using Cu-K_α_ radiation (*λ* = = 1.5406 Å) in the range of 20°–80° at a scan rate of 0.05°/min. The surface morphology was investigated using SEM (JSM-IT800 NANO SEM, Japan) with an accelerating voltage range of 0.01–30 kV and an Oxford X-MaxN 50 mm^2^ silicon drift detector (Oxford Instrument, UK) was used for EDX analysis. The SAED and particle image were evaluated using a TEM (JEOL-2100) at 200 kV. Thermofisher Evaluation 220 spectrophotometer was used to analyze the UV–visible absorbance studies. A Xenon lamp was utilized as the excitation source (Oxford low-temperature LN2 77 K setup) for the photoluminescence (PL) measurement, which was performed using a Varian Cary Eclipse spectrometer set to 325 nm as the excitation wavelength. Current-Voltage measurements were conducted via Keithley meter (Model No. 6517 B) with temperatures ranging from 30 to 150 °C, with the assistance of a temperature controller.

### 2.4 Electrochemical analysis

PEC studies were performed via the linear sweep voltammogram (LSV) approach with simulated solar light calibrated at 56 mW/cm^2^ on the film surface Electrochemical measurements were performed in potentiostat/galvanostat (Bio-Logic SP-300) with three-electrode arrangement is used for PEC water splitting, with Ag/AgCl serving as the reference electrode. The reversible hydrogen electrode (RHE) is mentioned in the PEC data reported in this context. Photoelectrode stability was analysed by chronoamperometry technique for 300 s. The EIS obtained in the frequency range of 0.1 Hz–100 kHz using a moderate AC stimulus of 10 mV. All electrochemical measurement was carried out three-electrode setup in a 0.5 M Na_2_SO_4_ electrolyte with a pH of 5 ± 0.1.

## 3 Result and discussions

### 3.1 Film thickness

The growth of CuO thin film with various pH levels by SILAR method can be controlling two separate variables such as (i) homogeneity and width of the film (ii) surface morphology [58,59]. The presence of hydroxyl ions in the solution used for precursors is essential for producing thin films with exceptional quality [60,61]. Stylus profilometry was employed to determine the films' thickness after they were deposited. As depicted in Fig. 1(b), the variation of the film thickness with deposition time for CuO thin films prepared at various pH values. It is noted that the film its thickness improves with the number of dips and reaches its maximum thickness obtained for the deposition process at the 20th dipping. The measured maximum film thickness was found to be ∼2.7 ± 0.02 μm, ∼1.8 ± 0.02 μm and ∼1.4 ± 0.02 μm also each dipping the film thickness was attained ∼66 nm, ∼93 nm, and ∼138 nm with pH 8.5 ± 0.1, 9.5 ± 0.1 and 10.5 ± 0.1 respectively. According to this result, it is evident that at the pH of 8.5 ± 0.1, there is a notable increase in the formation of the gel state in the copper acetate solution. Simultaneously, the concentration of unreacted Cu^2+^ ions is higher under these conditions. Consequently, during the dipping of the ITO substrate into the solution, a greater volume of the solution tends to deposit on the substrate surface, resulting increase in film thickness value. Further increasing the pH value up to 9.5 ± 0.1 and 10.5 ± 0.1 the Cu^2+^ ion concentration will be lower and OH^−^ ions may compete with Cu^2+^ for binding sites forming insoluble Cu (OH)_2_ and reducing the concentration of free Cu^2+^ available for deposition process. The reduction in ion concentration leads to a decrease in the rate of the deposition of ions on the surface of the substrate, resulting in a decrease in film thickness [62,63].

### 3.2 Structral properties

The XRD pattern of non annealed thin film at different pH values in between 8.5 ± 0.1 and 10.5 ± 0.1 as shown in Fig. 1(c). From this figure we observed three noticeable peaks 2θ values around at 23°, 35° and 38° are corresponding to (021) (002) and (022) planes with orthorhombic structure of Cu(OH)_2_ (JCPDS card no. 72–0140), Fang et al. [64]. The broad hump peak that appears at ∼23° indicates that the material which was deposited as amorphous in nature, consistence with earlier reports [65,66]. In Fig. 1(d) displays XRD array of CuO thin films calcined at 400 °C. This XRD pattern analysis shows that the film deposited at pH 8.5 ± 0.1 has some noticeable peaks along with more noise peaks, which suggests poor crystallinity. This result caused by the lower pH during deposition, which limits particle growth and by the significant amount of unreacted Cu^2+^ ions in the solution. The pH value increasing to 9.5 ± 0.1 and 10.5 ± 0.1, the XRD peaks seems to be very sharp indicating crystalline phase. This result indicates that an increase in pH values can be attributed to the substantial reaction of Cu^2+^ ions with ammonium hydroxide, resulting in the generation of a larger amount of Cu (OH)_2_ and consequently achieving well crystallinity. The process of the calcination leads to produce CuO thin films from Cu (OH)_2_ [67,68]. In accordance the reflected peak 2θ values at 32.52, 35.54, 38.76, 48.82, 53.48, 58.20, 61.54, 66.31, 68.02, 72.45, 75.23 and 82.59 are corresponding to the (110), (11-1), (111), (20-2), (020), (202), (11-3), (31-1), (220), (311), (22-2) and (31-3) planes with monoclinic crystalline CuO (JCPDS: 00-005-0661) [69,70]. The size of crystals that form over the substrate's surface is known as the crystallite size (D), and it can be determined using Scherrer's Eq. (5).

(5) $$D=\frac{k\lambda }{\beta \;\cos\;\theta }$$

where, k is the Scherrer constant (typically around 0.9), *λ* is the X-ray wavelength (Cu Kα = 1.5406 Å), θ is diffraction angle and β is the full width half maximum in radian. The crystallite size was determined based on the prominent orientation plane (111), resulting in calculated sizes of 19.24 nm, 22.33 nm, and 25.62 nm for films deposited at pH values of 8.5 ± 0.1, 9.5 ± 0.1, and 10.5 ± 0.1, respectively. The observed increase in crystallite size with pH indicates the nucleation and growth is highly occurrence at the pH value 10.5 ± 0.1 and the unreacted Cu^2+^ ions is very less resulting increasing of crystallite size. Similar studies indicated that pH had an effect on the crystallinity of the produced film [39]. The most prominent intensity (111) plane is found to increase with pH value from 8.0 ± 0.1 to 10 ± 0.1 indicating the prepared film grown in the (111) orientation. The term “d" refers to the lattice spacing, which is the inner planer spacing among atom arrangements in the specified plane and can be determined using Eq. (6). The measured “d" are close agreement with the JCPDS file (00-005-0661) for monoclinic with lattice parameters (a = 4.59 Å; b = 3.53 Å; c = 5.34 Å) and the values are summarized in Table 1.

(6) $$\frac{1}{d^{2}}=\frac{1}{\sin^{2}\;\beta }\left(\frac{h^{2}}{a^{2}}+\frac{k^{2}\;\sin^{2}\;\beta }{b^{2}}+\frac{l^{2}}{c^{2}}-\frac{2hl\;\cos\;\beta }{ac}\right)$$

**Table 1:** Structural parameters of CuO thin films deposited at different solution pH values.

Structural parameters	pH 8.5 ± 0.1	pH 9.5 ± 0.1	pH 10.5 ± 0.1
FWHM (111) plane	0.45	0.39	0.34
Crystallite size, D (nm) (111)	19.24	22.33	25.62
Texture co-efficient (TC)(111)	0.34	0.41	0.47
Dislocation density, δ (nm^−2^)	2.2701 × 10^−3^	2.005 × 10^−3^	1.523 × 10^−3^
Microstrain, *ε* (line^−2^ m^−4^)	5.58 × 10^−3^	4.84 × 10^−3^	4.22 × 10^−3^
Lattice constant (Å)	a = 4.59; b = 3.53; c = 5.34	a = 4.61; b = 3.40; c = 5.28	a = 4.65; b = 3.41; c = 5.25
Atomic radius (r) (Å)	2.2954	2.3051	2.3257
Volume of the particle (V_p_) (Å^3^)	48.58	49.22	50.51
Volume of the unit cell (V_u_) (nm^3^)	85.54	81.35	80.74
Atomic packing factor (APF) (%)	56	60	61

**Fig. 2:**
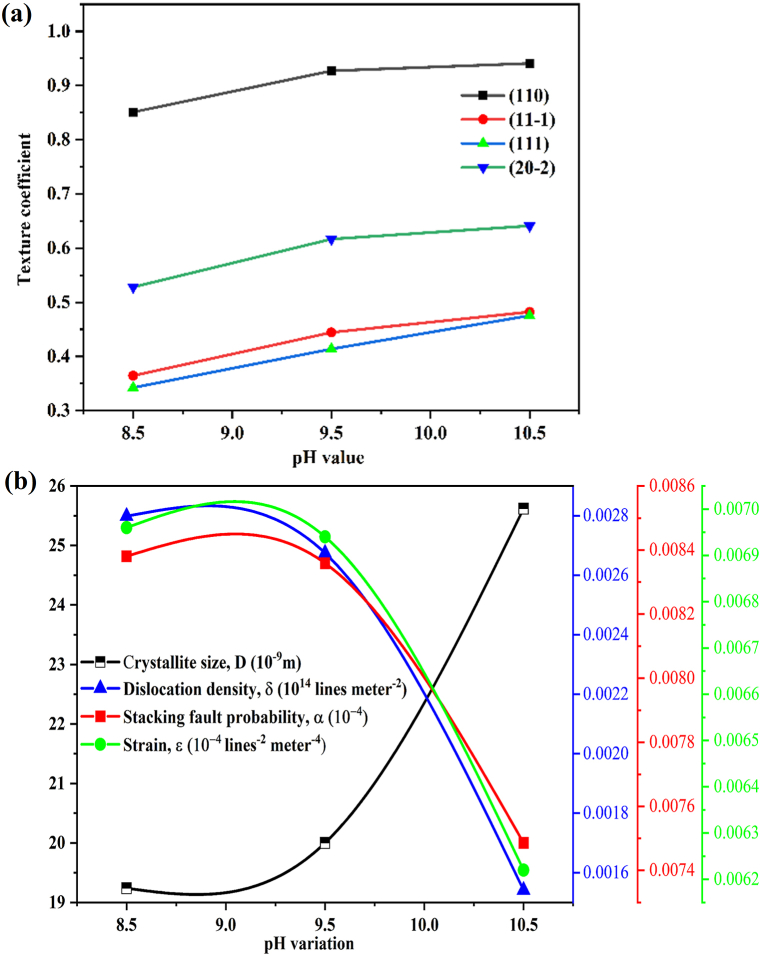
(a) Texture coefficient and (b) variation of structural parameter with crystallite size for CuO thin film at different pH values 8.5 ± 0.1, 9.5 ± 0.1 and 10.5 ± 0.1.

The texture of a given plane is indicated by its texture coefficient, TC_(*hkl*)_ of plane; the plane's deviation from the conventional sample indicates the preferred growth. The texture of the developing material is directly influenced by the film thickness. Eq. (7) was utilized for determining the texture coefficients.

(7) $$TC=\frac{I_{\left(hkl\right)}/I_{0\left(hkl\right)}}{\frac{1}{N}\sum_{N}I_{\left(hkl\right)}/I_{0\left(hkl\right)}}$$

where, I_(*hkl*)_ is the measured intensity for specific plane, I_0(*hkl*)_ is the standard intensity for specific plane, and N is total number of diffraction peaks obtained. The T_C(*hkl*)_ values should be lower than 1 to indicate a preferential orientation of the (111) plane [39]. Texture coefficients above 1 typically indicate a microcrystalline nature in materials, while a value of zero suggests amorphous structure, indicating a lack of defined orientation. Values below 1 often correspond to nanocrystalline materials. Using texture coefficient analysis, the positive and negative notation plane texture values are increasing with pH values as shown in Fig. 2 (a). These results are consistent with both the increased intensity of XRD peaks and the observed increase in crystallite size. There is a consistent trend in the structural features of the prepared samples, as evidenced by the texture coefficient analysis, larger crystallite sizes and higher intensity XRD peaks. The obtained CuO thin film texture coefficient value are consistent with earlier reports Gençyılmaz et al. [39] & Daoudi et al. [55]. Structure and morphology are strongly affected by dislocations and strain. The morphological and optical characteristics of thin films are substantially influenced by defect concentration. The number of dislocation of atoms per unit volume is known as dislocation density (δ) also, dislocation of atoms was decrease with increasing of crystallite size [71]. Micro-strain (*ε*) is a measure of the local distortions or imperfections within the crystal lattice of a material [72]. The following Eq(s). (8) & (9) was used to calculate the value of dislocation density and lattice strain of the prepared thin films.

(8) $$\delta =\frac{1}{D^{2}}$$

(9) $$\varepsilon =\frac{\beta_{hkl}}{4\;\tan\;\theta }$$

A crystal's strain value indicates its alignment. When a film was deposited, the film's crystalline strain was identified by comparing the diffraction pattern with the line broadening that came from XRD measurements [[67], [73], [74]]. All diffracted angles of the deposited CuO film contributes to ***β***_h*kl*._ The total peak broadening, represented by the following Eq(s). (10) & (11) represents a combination of the size of the crystallite and material strain.

(10) $$\beta_{hkl}={\left[{\left(\beta_{hkl}^{2}\right)}_{\text{measured}}-{\left(\beta_{hkl}^{2}\right)}_{\text{standard}}\right]}^{1/2}$$

(11) $$\beta_{hkl}=\beta_{a}+\beta_{b}$$

where, ***β***_*a*_ and ***β***_*b*_ are the effects of size of crystallite and strain-induced broadening, respectively.

and ***β***_h*kl*_ denotes FWHM of the instrumentally adjusted broadening. The strain value and the crystallite size are independent parameters. The conditions of the experiment (instrument-dependent) and the properties of the treated sample interact in a complex way to estimate the Bragg peak's breadth. The equations mentioned earlier, specifically Eq(s). (5) & (9), are combined into the following form, expressed as Eq. (12).

(12) $$\beta_{hkl}\;\cos\;\theta =\frac{k\lambda }{D}+4\varepsilon \;\sin\;\theta$$

**Fig. 3:**
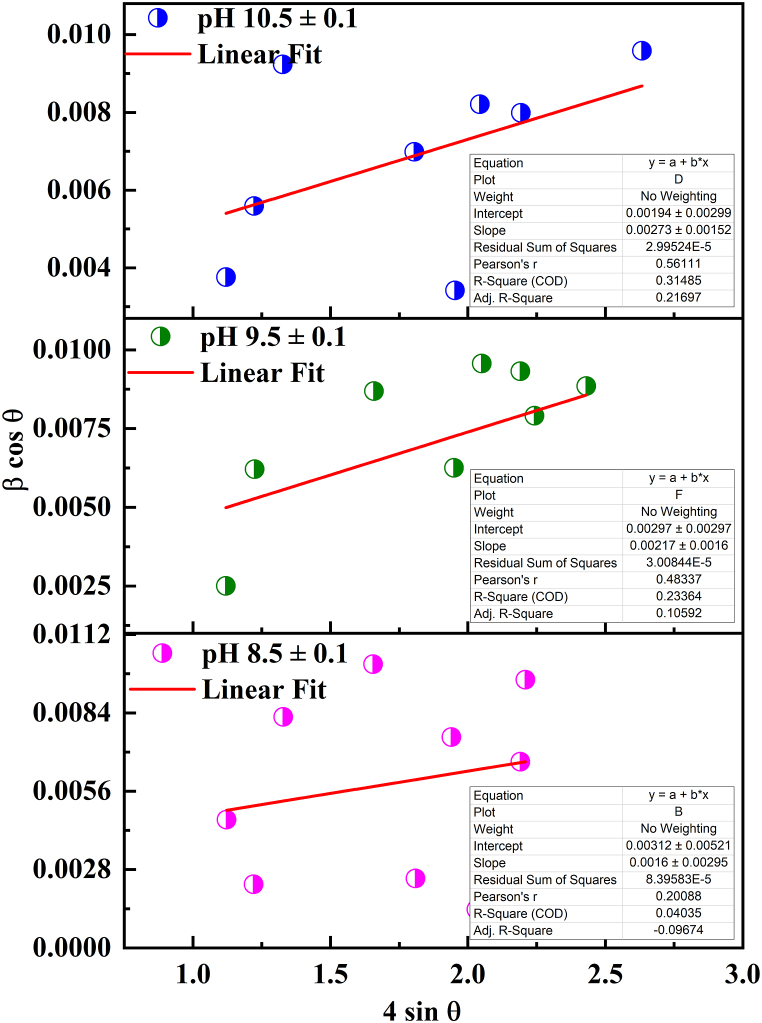
Williamson Hall plot for CuO thin film at different pH values 8.5 ± 0.1, 9.5 ± 0.1 and 10.5 ± 0.1.

In order to solve Eq. (12), it is assumed that the crystalline strain is uniform across all crystallographic orientations. In the Williamson-Hall plot, the slope of the plot is related to the reciprocal of the average crystallite size (1/D), and the intercept provides information about the strain in the crystal lattice. While the W–H method uses tan θ, the Scherrer-equation follows a 1/cos θ dependency. The fundamental distinction was that the reflection broadening caused both microstrain and small crystallite size, which are caused by microstructural factors. The separation of size and strain broadening analysis is carried out using Williamson and Hall, depending on various θ positions. Prabhu et al. [75] reported similar investigation for separation of size and strain broadening analysis. In Fig. 3, a plot of 4 sin θ versus β cos θ is presented for CuO thin films at different solution pH levels. Under this assumption of uniform crystalline strain, a uniform deformation model (UDM) is employed. The UDM posits that all crystals exhibit isotropic behaviors, meaning their characteristics are independent in all directions along their dimensions, resulting in a lack of strain in the produced film. The fitted line's slope and y-intercept are used to compute the strain and particle size, respectively. It was noted that this strain might be caused by lattice shrinkage based on the computations of lattice parameters. The stacking fault probability (α) represents the proportion of layers experiencing stacking sequence faults within a specific crystal and hence one fault is expected to be found in 1/α layers [73]. The presence of stacking faults gives slight shift in the reflected peak position with respect to ideal positions. The value of stacking fault probability (α) was measured by using Eq. (13).

(13) $$\alpha =\left[\frac{2\pi^{2}}{45{\left(3\;\tan\;\theta \right)}^{1/2}}\right]\beta$$

The variation in “D”, “δ”, and “*ε*” and “α” for CuO thin films with three different solution pH values are depicted in Fig. 2(b). From this observation the microstructural values are smaller than crystallite size and the measured values are summarized in Table 1. The size of an atom is determined by its atomic radius (r), which is commonly measured as the separation between nucleus and electron clouds [76]. The atomic packing factor (APF), also known as packing efficiency or packing fraction, is a dimensionless quantity that represents the fraction of space within a crystal structure that is occupied by atoms [77,78]. The atomic radius and atomic packing factor of fabricated CuO thin film was measured by using Eq.(s) (14) & (15). The measured structural parameter variation values are given in Table 1.

(14) $$r=\frac{1}{2}a$$

Number of particles in the unit cell, N = 1 and volume of the particle (V_p_) (Eq. (16)) and volume of the unit cell (V_u_) (Eq. (17)) is determined atomic packing factor (APF).

(15) $$APF=\frac{NV_{P}}{V_{u}}=\frac{\pi a^{2}}{6bc\;\sin\left(\beta \right)}$$

(16) $$V_{p}=\frac{1}{6}\pi a^{3}$$

(17) $$V_{u}=abc\;\sin\left(\beta \right)$$

### 3.3 Morphology and film composition

**Fig. 4:**
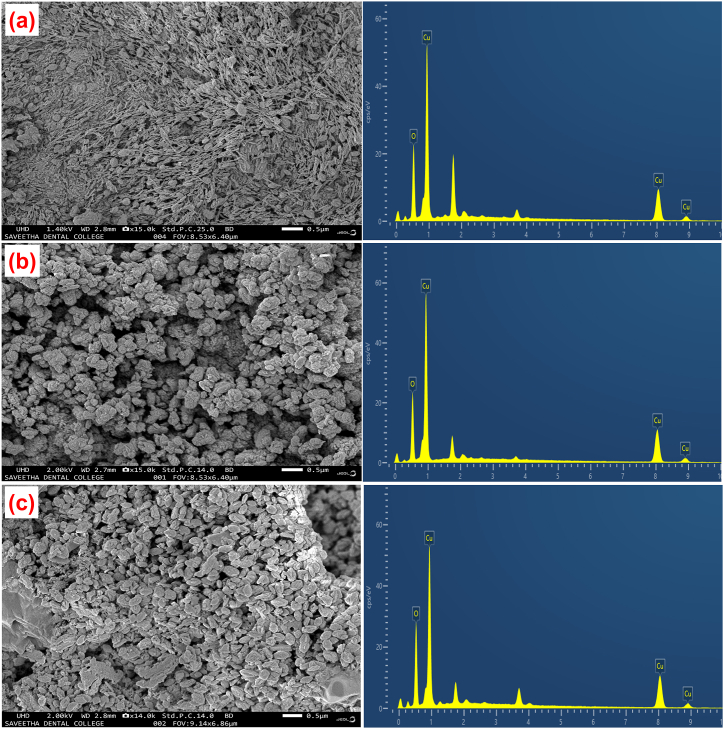
SEM images with EDX pattern of CuO thin films deposited at different pH values: (a) 8.5 ± 0.1, (b) 9.5 ± 0.1 and (c) 10.5 ± 0.1.

The SEM images with EDX of prepared all CuO thin films with various pH values (pH = 8.5 ± 0.1, 9.5 ± 0.1 and 10.5 ± 0.1) as shown in Fig. 4(a–c). From this figure, the CuO thin films produced at solution pH value 8.5 ± 0.1 is found to exhibit agglomerations of different shaped like spherical and rod shaped particle as shown in Fig. 4 (a). But the CuO thin film produced pH value at 9.5 ± 0.1 (Fig. 4 (b)) is seems to be slight variation of uneven spherical particle formation compared with pH value at 8.5 ± 0.1. The presented spherical particle at a film-deposited pH value of 8.5 ± 0.1 is consistent with the film-deposited pH value of 9.5 ± 0.1, suggesting the development of agglomeration or chain length growth within the pH range of 8.5 ± 0.1 to 9.5 ± 0.1. Further increasing the pH value to 10.5 ± 0.1 the formed particle is more clearly with individual particle identification as shown in Fig. 4 (c). This result suggests that variations in pH have a notable impact on the nucleation and growth of CuO grains in each SILAR cycle. An increase in pH appears to contribute to more controlled and uniform processes, influencing nucleation sites and growth rates. This, in turn, leads to the formation of more uniformly sized CuO grains [79]. As a result, the pH value at 10.5 ± 0.1 is enhanced the structural qualities will be positively impacted by this granular and apparent structure [80]. The elemental analysis result is clearly shows the presence of Cu and O elements for prepared all thin films and the atomic percentage of prepared thin films were shown inside of the EDX pattern. From this result, the film attained pH at 8.5 ± 0.1 and 9.5 ± 0.1 have near stoichiometry ratio whereas the pH at 10.5 ± 0.1 exhibit perfect stoichiometry ratio. This result suggesting increasing of pH values are controlled oxidation state of the prepared thin film.

### 3.4 Transmission electron Microscope

**Fig. 5:**
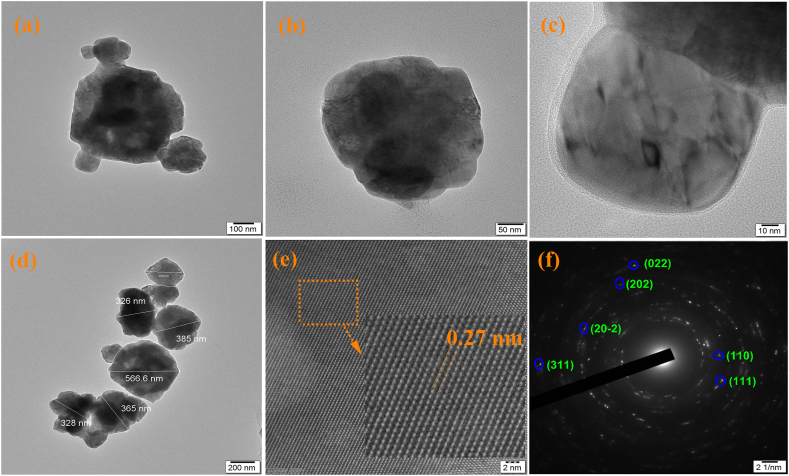
TEM (a–d) Different magnification particle images (200, 100, 50 and 10 nm), (e) Latic springes image, (f) SAED pattern for CuO thin film deposited at pH 10.5 ± 0.1.

The TEM particle image for CuO thin film prepared at pH 10.5 ± 0.1 were shown in Fig. 5. The particle are well separated with different magnification as shown in Fig. 5(a–d). The 200 nm scale observation seems to be the average particle size in the range between ∼200 nm and 300 nm. The higher magnification 100 nm, 50 nm and 10 nm view is clearly exhibit, one particle is bonded with another particle. The SAED pattern of bright circular rings is belongs to (110), (111), (202), (022), (20-2) and (311) planes of CuO were shown in Fig. 5 (f). The lattice spacing values of CuO thin film prepared at pH 10.5 ± 0.1 was found to be 0.27 nm as shown in Fig. 5 (e). According to this TEM SAED and lattice fringes observation the prepared film have polycrystalline nature.

### 3.5 Optical properties

**Fig. 6:**
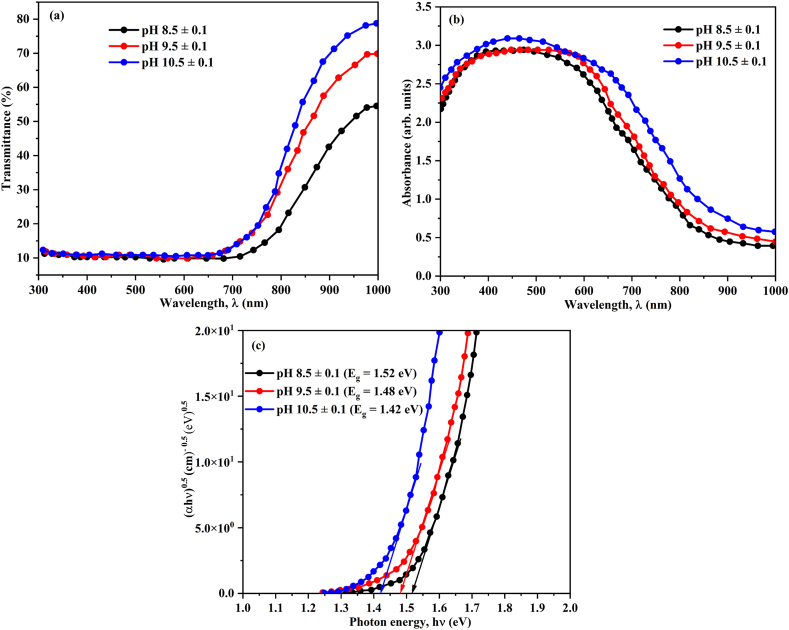
(a) Transmittance (%), (b) UV–vis Absorbance graph and (c) Tauc plot for CuO thin film deposited at different pH values.

**Fig. 7:**
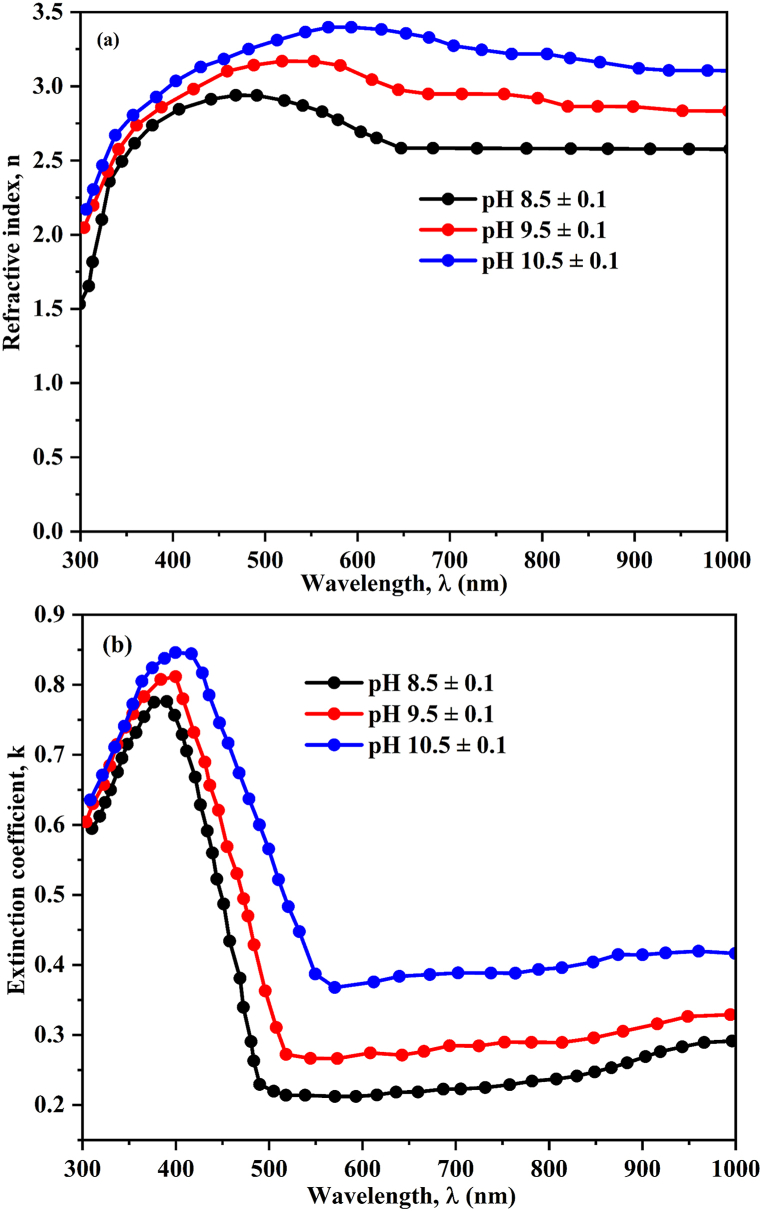
Variation of (a) Refractive index (n) and (b) Extinction co-efficient (k) with wavelength of the prepared CuO thin films at different pH values.

Analyzing incident light passage through prepared materials is an important way to evaluate their spectral properties. Moreover, modifying either the intensity or propagation vector of the incident wave can cause variation in the optical properties of thin films, consequently influencing g the spectrum of incident light as it traverses through the films [81]. The optical characteristics of all CuO thin films were examined in the wavelength range of 300–1000 nm. The following Eq(s). (18) & (19) provides the measured values of the reflectance, R_exp_ (*λ*) and transmittance, T_exp_ (*λ*), after correction.

(18) $$T\left(\lambda \right)=\left(\frac{I_{fs}}{I_{s}}\right)\left(1-R_{s}\right)$$

(19) $$R\left(\lambda \right)=\left(\frac{I_{fr}}{I_{M}}\right)\left[1+{\left(1-R_{s}\right)}^{2}\right]R_{M}-T^{2}R_{s}$$

where, I_fs_ and I_s_ represent the intensities of light passing through the film-substrate (ITO) and reference substrate system respectively. R_s_ denotes the subtract reflectance. Furthermore, I_fr_ denotes the intensity of incident photon reflected from CuO film sample, whereas I_M_ and R_M_ represent the intensity of intensity photon reflected from the reference mirror and the reflectance mirror respectively. Fig. 6(a) depicts the spectral behaviour of the corrected transmittance (*λ*) for the CuO thin films prepared at various pH values. All samples were found to be highly transparent upon the band edge in the NIR spectral region. Furthermore, the transmittance percentage of fabricated CuO films pH values at 8.5 ± 0.1 and 9.5 ± 0.1 seems to be lower compared than pH value 10.5 ± 0.1 due to densely grown the CuO particle on surface of the substrate. According to Beer-Lambert's law (T = e^−αt^). the CuO thin film deposited at pH 10.5 ± 0.1 exhibits higher transmittance percentage, which can be explained by its semiconductor behavior and thinner sample. Photovoltaic applications can benefit from the CuO thin film's greater transparency [82]. Furthermore, as solution pH increased, the optical absorption edge shifted towards longer wavelengths. This may be due to the reason of raising the pH value increases the kinetic energy of molecules, resulting to their rearrangement, reduction in disorder, decreasing of vacancies and thus increasing of the grain size [83]. Raising the pH value of the solution leads to beneficial effects such as minimizing defects in the texture of the films. As a result of having less defects, the film takes on properties of becoming thinner, lighter in weight and more transparent [84,85]. All samples T (*λ*) spectra can be separated into two specific spectral regions: (1) a strong-absorption region where *λ* is usually less than 800 nm, and (2) a high transparent region where *λ* is usually between 800 nm and 1000 nm. Similar behavior was observed for other semiconductor metal oxide thin film in previous report, Hassanien et al. [81]. The solution pH value improves, the absorption peak shifts is slightly varied as shown in Fig. 6 (b) [86]. The selection of semiconducting materials for prospective applications is significantly influenced by the optical absorption spectra. Furthermore, analysing these spectra provides details about bandgap (E_g_) of the materials and its nature of electronic transitions. The coefficient of absorption (α) with wavelength will be investigated using T(*λ*) and R(*λ*). The absorption coefficient (α) can be measured using the following Eq. (20).

(20) $$\alpha \left(\lambda \right)=\frac{1}{t}.\ln\left[\frac{{\left(1-R\right)}^{2}}{2T}+\sqrt{\frac{{\left(1-R\right)}^{4}}{4T^{2}}+R^{2}}\right]$$

where, (t) is the CuO film thickness (Measured thickness values in nanometre: pH 8.5 ± 0.1 = 2700 × 10^−9^, (pH 9.5 ± 0.1 = 1800 × 10^−9^ and pH 10.5 ± 0.1 = 1400 × 10^−9^). Moreover, the present CuO films absorption edge shifted towards higher wavelengths when the pH was raised from 8.5 ± 0.1 to 10.5 ± 0.1 and its band edge located in the visible region for all films. Notably, the thin-film sample with the highest pH (pH = 10.5 ± 0.1) has an extremely wide band edge. The absorption edge shift may be caused by a number of factors, including an increase in crystallite size, an increase in atom agglomerations, a decrease in disorders, and a decrease in localized state density. These obtained results are consistent with other semiconducting crystalline thin films [81,[87], [88], [89]]. To determine the optical band-gap energy and nature of transition in the deposited CuO film is based upon the “α” with respect to “hυ” in the strong-absorption region can be discussed in terms of Tauc's equation (Tauc et al. [90]), which is given as follows as following Eq. (21).

(21) $${\left(\alpha hv\right)}^{n}=\alpha_{0}\left(hv-E_{g}\right)$$

where “n” is the transition mode's power factor, “α_0_” is the band tailing variable. The Mott and Davis density of localized state model [91] determines the optical energy gap, Eg, which lies among the localized states near the mobility edges. According to Koffyberg et al. [92], a further direct allowed ((αhυ)^2^ on hυ) interband transition occurs at 3.25 ± 0.05 eV for CuO nanoparticles. The linear relationship of (αhυ)^1/2^ on hυ in the broad wavelength region indicates an implicitly allowed band gap of 1.35 ± 0.02 eV. Plotting (αhυ)^n^ vs. hυ for prepared CuO thin films with varying power values revealed that the plots with n = ½ and indirect bandgap are the most appropriate and suitable. The curve's linear portion to be extrapolated to the axis of absorption were illustrated in Fig. 6 (c) inset. From this figure, the CuO allowed indirect optical transitions, i.e., (αhυ)^0.5^ vs. (hυ). The measured energy gap (eV) was found to be 1.52 eV, 1.48 eV and 1.42 eV with indirect transition for film deposited pH values at 8.5 ± 0.1, 9.5 ± 0.1 and 10.5 ± 0.1 respectively. Furthermore, the Daoudi et al. [55] reported this same behavior for reduction of bandgap (E_g_) value with increasing of crystallite size for CuO thin film prepared by SILAR method. The determined E_g_ values are summarized in Table 2. It was observed that Eg as decreased with increasing pH value of the CuO thin films. The decreasing of energy gap value with increasing pH due to the shift of absorption band edge was slight variation towards with visible region. Also, the pH increasing, the crystallinity was increased, there results are confirmed earlier findings like structural parameter analysis (Texture coefficient, dislocation density, strain and particle agglomeration). As a result, the structural and optical properties of the prepared CuO thin films lead to an increase in the pH value of the solution. Furthermore, the direct energy gap is higher than the indirect energy gap the materials will be crystalline. The pure, CuO direct energy gap value is in the range between 3.50 eV and 3.85 eV whereas, indirect energy gap value is in the range of 1.2 eV–1.4 eV. Our measured energy gap value is consistent with indirect transition and these obtained values are consistent with earlier reports [[36], [37], [38],^55^,^92^]. The variation of the refractive index “n” as well as the extinction coefficient “k” with wavelength in the range of 300 nm–1000 nm are shown in Fig. 7 (a & b). The refractive index is directly related to the speed of light in the material resulting, refractive index increasing the speed of the light value is decreases which is measured using Eq. (22) [39].

(22) $$n=\frac{1+R}{1-R}-\sqrt{\frac{4R}{1-R}-k^{2}}$$

**Table 2:** Optical parameter values of CuO thin films deposited at different solution pH values.

CuO sample pH (±0.1)	Film thickness (±0.02 μm)	Energy gap (Eg) (±0.2 eV)	Refractive index (n)	Extinction coefficient (k)
pH = 8.5	2.5	1.52	2.8	0.76
pH = 9.5	1.8	1.48	2.9	0.77
pH = 10.5	1.4	1.42	3.2	0.82

The spectral dependence estimated refractive index (n) with wavelength of the current CuO thin films as shown in Fig. 7(a). The refractive index is higher for all thin-film samples and at wavelengths less than 550 nm in the strong-absorption region. The observed result is caused by the resonant effect of incident electromagnetic radiation interacting with electronic polarization. The interaction between electrons in CuO thin films and the oscillating electric field leads to the peak values of “n” occurring precisely at energy levels corresponding to the forbidden energy gaps. This phenomenon is indicative of thin film polarization dynamics. Moreover, the polarization within these films consistently maintains high levels, contributing to the reduction in electromagnetic wave velocity and consequent elevation of the refractive index [81,93]. The refractive index decreases sharply at longer wavelengths (550 nm ≤ *λ* ≤ 1000 nm). Furthermore, the current CuO thin films behave similarly to a normal dispersion in terms of refractive index versus wavelength and the value of (n) was found to increase as the pH of the solution increased. Gencyılmaz et al. [39] found similar trends in CuO semiconductor thin films. The measured optical parameter value are summarized in Table 2. The extinction coefficient (k), often referred to as the absorption index, serves as a measure of the energy absorbed by a material upon penetration by electromagnetic waves. Consequently, it denotes a decrease in the intensity of electromagnetic waves that traverse the material. The values of the “k” are influenced by structural imperfections and the abundance of free electrons within the material. The “k” value was measured using Eq. (23).

(23) $$k=\frac{\alpha \lambda }{4\pi }$$

As shown in Eq. (23), the extinction coefficient (k) decreases with increasing wavelength (***λ***), affecting the materials light attenuation properties. Fig. 7 (b) shows that the extinction coefficient also decreases rapidly as the wavelength of the incident photon increases in the high absorption region up to about 1000 nm. Following this wavelength, the k value nearly became constant. A similar trend was observed in various partially crystalline and non-crystalline semiconducting materials [55,81,81,94].

### 3.6 Photoluminescence

**Fig. 8:**
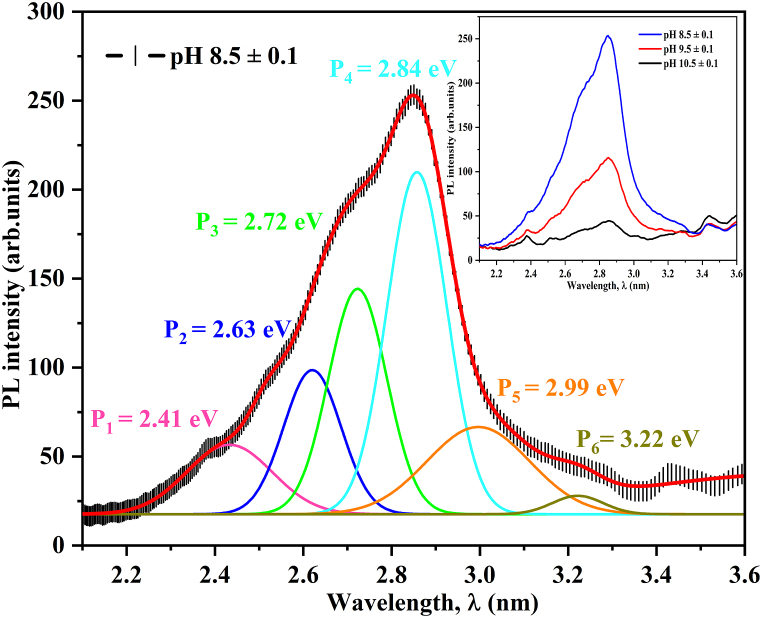
Photoluminescence spectra of CuO thin film deposited at different solution pH values.

The PL technique is well-known for its non-invasive nature and high sensitivity in analyzing the electronic structure and defects of various materials. However, it is critical to recognize the scarcity of literature on the PL properties of CuO thin films. Despite the existence of numerous studies, a significant portion fail to provide comprehensive explanations for the observed luminescent properties, owing primarily to the intricate band structure inherent in CuO. In line with this, our current study reveals unconventional emissions in CuO thin films, adding to the complexity of the observed phenomenon. In Fig. 8 shows the PL emission spectra of a CuO thin film fabricated with solution pH ranging from 8.5 ± 0.1 to 10.5 ± 0.1. All PL measurements were made at room temperature with a 325 nm laser for excitation and targeting the conduction band of all fabricated films. The analysis revealed the presence of several distinct transitions (labeled P_1_–P_6_) in the PL spectrum. This figure shows a near-band-edge emission (NBE), which occurs when an electron transitions from conduction band to valence band. This intrinsic emission phenomenon has also been reported in studies by Zheng et al. [95] and Hashim et al. [96], which focused on ZnO UV emission in the 370–390 nm range. Furthermore, these emissions can be caused by shifts in imperfect energy states or between levels of defect are also known as defect-related emissions. The NBE emissions typically coincide with or possess energy levels near the bandgap energy, whereas defect-related emissions have significantly lower photon energy. To determine the emission photon energy of CuO thin films, Gaussian curve fitting was used under 325 nm excitation to precisely determine peak positions. The PL spectra show peaks labeled P1–P6 at different photon energies. The peak labeled P1, observed at 2.41 eV (∼514 nm) in the CuO thin film, is associated with green emission and could be related to a few steps: (i) electronic shifts in single ionized oxygen gaps along with photo-excited electrons, or shifts related to electrons close to the region of conduction, (ii) completely trapped electrons at single charged oxygen gaps and surface imperfections and (iii) band-to-band transitions within CuO [97]. The P_2_ peak at 2.63 eV (∼471 nm), which indicates blue emission, is caused by a shift in electronics from the acceptor energy state of oxygen interstitial to the donor energy state of copper vacancies. These emissions could result from electron shifts between defect levels inside of the forbidden band. However, the violet emission does not appear to originate from a direct electron transition between the bottom of the conduction band and the top of the valence band, as the photon energy of the violet emission exceeds the bandgap value (1.42 < 2.63 eV). Similar incidents have been reported in other research studies. As an example, Al-Ghamdi et al. [98] used magnetron sputtering to create three CuO thin films, all of which emitted UV light at 387 nm (3.20 eV), with bandgaps of 1.73 eV, 1.88 eV and 2.2 eV. They did not specify the source of the UV emission. Chand et al. [99] used a pulsed laser to deposit Li-doped CuO thin films. At 325 nm UV excitation, these CuO thin films showed similar emission spectra. They all had three luminescent centers: UV emissions at 359 and 383 nm, and blue emission at 441 nm. The Li-doped CuO thin film's bandgap decreased from 2.99 eV to 2.76 eV as the Li doping concentration increased. Chand et al. concluded that the UV emissions centered at 359 and 383 nm were CuO band edge emissions, whereas the blue emission centered at 441 nm was caused by defects such as oxygen vacancies, etc. Whereas this unusual luminescence behavior is uncommon in other metal-oxide semiconductors, it has been observed several times in CuO nanomaterials, indicating that CuO's band structure is unique. The violet emission peaks P_3_ at 2.72 eV (∼455 nm) and P_4_ at 2.84 eV (∼436 nm) are caused by a non-radiative transition from the conduction band's sub-band to oxygen interstitial states [100]. The presence of peaks P_5_ at 2.99 eV (∼414 nm) and P_6_ at 3.22 eV (∼385 nm) in the emission spectra is indicative of near band edge emission or inter-band emission. These peaks are associated with electron-hole pair (excitonic) recombination processes. The demonstration of a broad violet to UV peak range in the spectra suggests that the as-deposited films exhibit crystalline characteristics with the desired optical properties. Furthermore, the pH increasing from 8.5 ± 0.1 and 10.5 ± 0.1 the PL intensity was decreased indicating the e^−^/h^+^ recombination is minimum compared other CuO thin films. Furthermore, our observed minimum PL emission intensity was found to be pH 10.5 ± 0.1 because the film thickness is very less compared other pH values. Similar results, Xu et al. [101] & Al-Ghamdi et al. [98] was reported. As the thickness decreases, the available volume for charge carriers (e^−^/h^+^) decreases, leading to a reduction in the number of excitons (e^−^/h^+^ pairs) that can be generated by photoexcitation. Therefore, the observed decrease in PL intensity with decreasing thin film thickness can be attributed to the reduced number of e^−^/h^+^ pairs generated and/or increased non-radiative recombination processes within the material.

### 3.7 Electrical properties

**Fig. 9:**
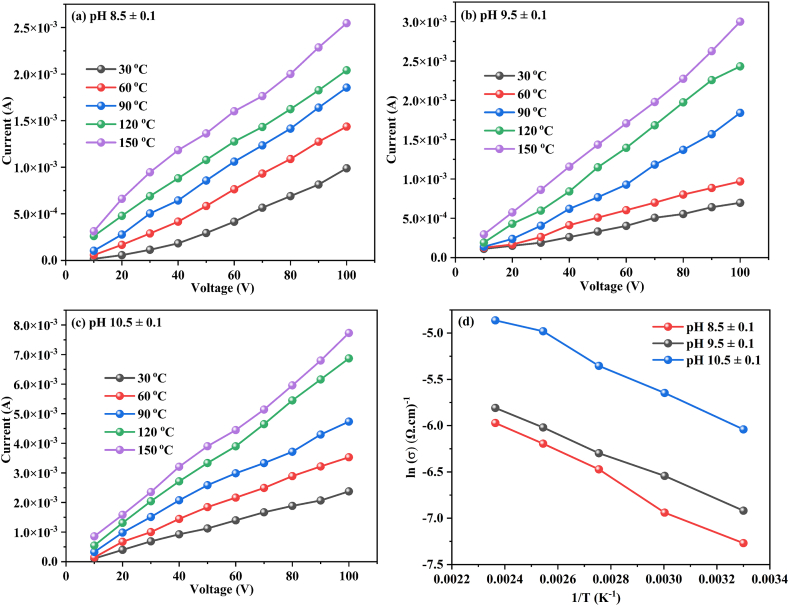
I–V characterizatics of the CuO thin films deposited at different pH values (a) 8.5 ± 0.1, (b) 9.5 ± 0.1, (c) 10.5 ± 0.1 and (d) Arrhenius plot of ln (σ) vs. 1/T for different solution pH values.

The DC electrical conductivity (σ_dc_) of the prepared all CuO thin films at various temperatures was measured using a Keithley electrometer in a two-probe configuration. The flow of current through the film was monitored using a steady voltage ranging from 10 to 100 V, as shown in Fig. 9(a–c). The current values increased linearly as the measurement temperature of CuO films increased from 30 to 150 °C. The “σ_dc_” of the deposited all films was investigated by using Eq. (24) [102].

(24) σ=dctRA

As shown in Eq. (24), the “σ_dc_” is given by the ratio of the sample thickness to the product of the cross-sectional area (A) and the resistance (R). The electrical conductivity value gradually increased as CuO thin film was deposited at various pH levels within the range of 8.5 ± 0.1 and 10.5 ± 0.1. The maximum average “σ_dc_” was found to be σ_dc_ = 5.0484 × 10^−3^ (Ω cm)^−1^ for CuO film deposited at pH 10.5 ± 0.1 compared pH 8.5 ± 0.1 (σ_dc_ = 1.7870 × 10^−3^ (Ω cm)^−1^) and pH 9.5 ± 0.1 (σ_dc_ = 1.9753 × 10^−3^ (Ω cm)^−1^). According to this result, the film deposited at 10.5 ± 0.1 has well-defined surface morphology, stoichiometric ratio and presence of free carriers' exhibit higher electrical conductivity compared to conditions at pH 8.5 ± 0.1 and 9.5 ± 0.1. Fig. 9 (d) exhibits the Arrhenius plot of ln (σ) vs. 1/T for different CuO thin film deposited at pH value in between 8.5 and 10.5 ± 0.1. The ln (σ) of the films decreased linearly with respect to inverse temperature (1/T) obeying the Arrhenius law [103].

### 3.8 Photoelectrochemical properties

**Fig. 10:**
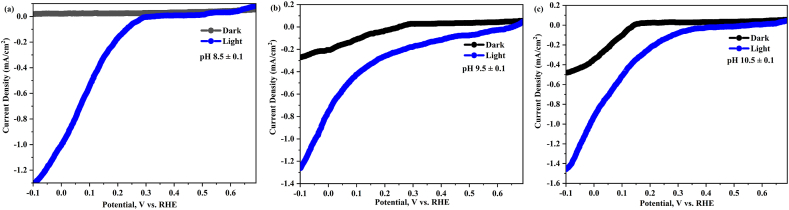
LSV curve under light and dark conditions for CuO photoelectrodes deposited at different pH values.

Copper oxide is commonly used in PEC cells for solar fuel generation due to its semiconducting properties and potential for PEC water splitting. However, there are several challenges associated with the stability of CuO during PEC, which can impact the overall efficiency and durability of the system. Franco et al. [104] reported CuO, a common electrode material, is susceptible to corrosion and decomposition under harsh conditions, particularly in PEC systems with electrolytes and high potentials. This leads to the deterioration of the electrode material over time, affecting its performance and stability. This degradation can significantly impact the performance and stability of the electrode over time. The degradation of CuO can be influenced by various factors, such as the operating conditions and initial electrode compositions. In the case of CuO as a conversion electrode for sodium-ion batteries, the complex nature of the electrode reaction and aging mechanisms can further complicate its application [105]. Lu et al. [106] reported the degradation behavior of CuO can also be affected by the stability of its phase composition, which is influenced by the electrolyte composition. However, the stability of CuO-based photocathodes in PEC systems can be improved by combining CuO with noble metal nanoparticles reported by Guo et al. [107]. Chaudhary et al. [108] found that irradiation-induced modifications in CuO thin films led to a decrease in PEC response, attributed to the creation of recombination centers. Similarly, Dengmet al. [109] demonstrated that a metal-organic framework coating on the surface of Cu_2_O enhanced its PEC performance by promoting charge separation and transfer. Prevot et al. [110] reported a high density of surface states in CuFeO_2_, which negatively impacted its PEC performance. Zhang et al. [111] showed that acceptor-doping in Cu_2_O improved its PEC performance by modifying its electronic structure. These vacancies can enhance PEC performance by promoting photoelectron activation and reducing electron-hole recombination Gan et al. [112], Singh et al. [113], Wang et al. [114]. However, the specific effects of oxygen vacancies on CuO include improving charge carrier concentration, facilitating charge separation and transfer, and enhancing PEC performance. Despite these advantages, oxygen vacancies can also act as charge recombination centers on the surface of CuWO_4_, leading to decreased efficiency in the generation of photocurrent [115]. Tang et al. [116] reported CuO's chemical instability in certain PEC cells can lead to the formation of unwanted by-products or surface degradation, compromising its stability. To address this, Ti alloying has been found to enhance CuO's stability in PEC water splitting, with a bilayer configuration of Ti-alloyed CuO on a pure CuO film achieving the best balance between stability and photocurrent. Similarly, the stability of Cu_2_O films has been improved by using p-type Cu_2_O films containing metallic copper inclusions, which change the films from n-type to p-type semiconductors Yang et al. [117]. However, the stability of Cu_2_O films in electrochemical cells can deteriorate over time, and the deposition of Au and SiO films onto Cu_2_O electrodes has been found to have mixed effects on stability and performance Khan et al. [118]. Another approach to improving stability is the embedment of anodized p-type Cu_2_O films with CuO nanowires, which enhances adhesion and passivates redox activities, leading to improved stability Wang et al. [119]. Photo-corrosion, a process where materials undergo accelerated corrosion under illumination, has been studied in various contexts. Xing et al. [120] found that a CuO photocathode modified with TiO_2_ and Pt displayed superior stability in photoelectrochemical water splitting. Burleigh et al. [121] observed that different metals, including pure copper, experienced photo-corrosion under ultraviolet light. Wang et al. [122] investigated the photocorrosion of a plasmonic enhanced Cu_x_O photocatalyst, identifying the reduction of Cu_2_O to Cu and the oxidation of Cu_2_O to CuO as the main causes. Beverina et al. [123] studied the phenomenon during post-chemical mechanical polishing cleaning of copper Damascene structures, finding that the use of certain inhibitors and the elimination of light during cleaning could mitigate the corrosion. To address these stability issues, researchers are actively exploring various strategies, such as the use of protective coatings, doping with other elements to enhance stability, and optimizing fabrication techniques to minimize surface defects. Additionally, the development of new materials with improved stability and efficiency is an ongoing area of research in the field of PEC water splitting. PEC measurements were performed in 0.5 M Na_2_SO_4_ using LSV method scan arate at 10 mV/s. In Fig. 10(a–c) shows the dark and light casing J-V curves (vs. RHE) for the produced photoelectrodes. In this study the CuO film deposited at different pH levels in between 8.5 ± 0.1 and 10.5 ± 0.1 utilized as the working electrodes, Ag|AgCl and platinum gauze as the reference electrode counter electrode respectively. In the presence of incident light on the surface of the working electrode, e−/h^+^ pairs are generated within the material. This generated pair undergoes separation driven by the built-in electric field existing at the electrode|electrolyte interface. Consequently, photoelectrons migrate towards the electrode surface, while the accompanying holes are concurrently directed towards the substrate, eventually reaching the back contact. This orchestrated movement completes an essential process in the conversion of light energy, facilitating the efficient generation of a photocurrent within the PEC system [1]. Photocorrosion in photoelectrodes is a prevalent phenomenon attributed to undesired charge kinetics at the electrode|electrolyte interface. Specifically, when photogenerated electrons, particularly in photocathodes, engage in reduction reactions, they can inadvertently reduce the electrode material, leading to photoreduction. This process of material degradation through corrosive reactions under light-induced conditions underscores the challenge of maintaining the stability and longevity of PEC systems. According to Eq. (25), the photocathodic generation of copper on the surface of the developed CuO photoelectrode is thermodynamically possible [3,124].

(25) $$CuO+2H_{2}O+2e^{-}\to Cu+2OH^{-}$$

**Fig. 11:**
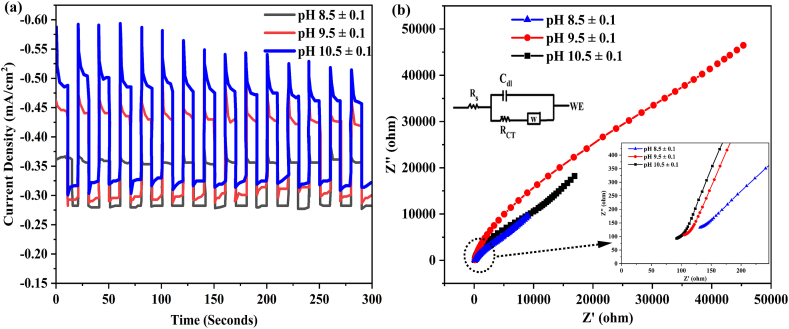
(a) Chronoamperometry curves of photoelectrodes under chopped illumination and (b) Nyquist plot for photoelectrodes for CuO photoelectrodes deposited at different pH values.

Under light illumination, the CuO photocathode demonstrates varying photocurrent densities depending on the pH during deposition. Specifically, the photocathode deposited at pH 10.5 ± 0.1 exhibits the highest efficiency, reaching a maximum photocurrent density of 1.45 mA/cm^2^ at −0.1 V vs. RHE. In comparison, photocathodes deposited at pH values 8.5 ± 0.1 and 9.5 ± 0.1 generate slightly lower photocurrent densities of 1.23 mA/cm^2^ and 1.27 mA/cm^2^, respectively. This data clearly indicates that the photocathode deposited at pH 10.5 ± 0.1 is the most efficient among the investigated conditions, establishing it as the optimal pH variation for achieving enhanced photocurrent performance in this study. In order to clarify the higher photoactivity, it is assumed that morphology plays a critical role in increasing CuO PEC activity. In Fig. 11(a) shows the chronoamperometric curve illustrates the performance of the constructed photoelectrodes, providing a comprehensive evaluation of their stability over a duration of 300 s under illumination conditions. From this figure the better photocurrent stability for the CuO film deposited at pH 10.5 ± 0.1 (70 %) as compared to pH 8.5 ± 0.1 (20 %) and pH 9.5 ± 0.1 (50 %). This indicates that the CuO photocathode deposited at pH 10.5 ± 0.1 exhibits enhanced stability, with substantially lower degradation of photocurrent over the evaluated time period, suggesting improved long-term performance under the specified experimental conditions. The investigation of charge transfer kinetics across the photoelectrode interfaces was carried out using Electrochemical Impedance Spectroscopy (EIS). The Nyquist plot, depicted in Fig. 11 (b), illustrates the impedance response of the deposited photoelectrodes under the analysis conditions of 0.5 M Na_2_SO_4_ at room temperature. The smaller semicircle was found to be electrodes deposited at pH 10.5 ± 0.1 compared to 8.5 ± 0.1 and 9.5 ± 0.1 indicating greater efficiency of charge separation and transfer across the CuO (pH 10.5 ± 0.1) electrode and electrolyte interface.

**Fig. 12:**
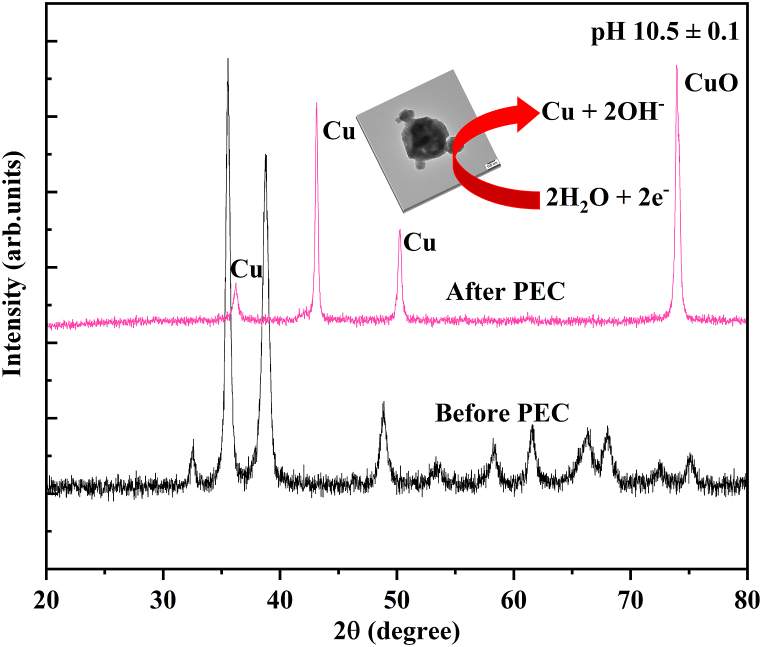
XRD pattern of CuO photoelectrodes prepared at the pH 10.5 ± 0.1: Before and After PEC.

The diffusion of Na^2+^ in the surface of the electrode material explained by an equivalent circuit model including solution resistance (R_s_), a charge transfer resistance (R_CT_), double layer capacitance (C_dI_) and Warburg impedance (Z_w_), which is similar to the circuit employed for the prepared all photoelectrodes. This results are determined by using Nova 1.01 software and measured values are summarized in Table 3. Among the characteristics provided, Rs indicates the resistance of the electrolyte, electrode substrate metal, electrode leads, terminals, and so on. The Rct denotes charge transport inside the cathode material. The R_s_ and R_CT_ value for CuO deposited at pH 10.5 ± 0.1 electrode were clearly the lowest compared with pH 8.5 ± 0.1 and pH 9.5 ± 0.1 indicating that the interfacial resistance was lowered and charge transfer at the electrolyte/electrode interface was considerably increased, as shown in Table 3. Eq. (26) was used to determine electrical conductivity of the prepared electrode [71].

(26) $$\sigma =\frac{d}{R_{b}A}$$

where, A is the area of the electrode, d is the thickness and R_b_ is the bulk resistance. The CuO (pH 10.5 ± 0.1) electrode material has high conductivity and a value of 0.6862 S/cm compared with CuO (pH 8.5 ± 0.1) (0.2779 S/cm) and CuO (pH 9.5 ± 0.1) (0.4646 S/cm) electrodes. An increase in electrical conductivity often corresponds to a decrease in the diffusion coefficient, especially for materials where charge carriers contribute significantly to both properties. The film deposited at pH 10.5 ± 0.1 exhibits a lower diffusion coefficient (1.036 × 10^−5^) compared to other samples (Table 3). This inverse relationship is due to densely packed charge carriers moving more freely, leading to greater resistance to diffusion as they are more likely to collide with other carriers. Finally, the after PEC analysis the XRD was performed for the CuO film deposited at pH value of 10.5 ± 0.1 were shown in Fig. 12. As a result, the observed peak at 2θ value 43.3 and 50.04 are related to the (111) and (002) plane of metallic Cu [3]. Eq. (25) suggested that the photoreduction formation of the Cu from the CuO surface is thermodynamically feasible.

**Table 3:** The value of R_s_, R_CT_, conductivity and diffusion coefficient (D_Na_^+^) for the prepared electrodes.

Electrode	R_s_ (Ω)	R_ct_ (Ω)	σ (S/cm)	D_Na_^2+^(cm^2^ s^−1^)
CuO (pH 8.5 ± 0.1)	7.24	440.35	3.16 × 10^−5^	2.35 × 10^−8^
CuO (pH 9.5 ± 0.1)	5.63	290.36	3.39 × 10^−4^	2.08 × 10^−8^
CuO (pH 10.5 ± 0.1)	3.57	190.44	3.49 × 10^−4^	1.93 × 10^−7^

## 4 Conclusion

The summary of this work, SILAR technique was used to produce a CuO thin film with three different pH values in between 8.5 ± 0.1 and 10.5 ± 0.1. The thickness of all the prepared CuO thin films with three different pH values (8.5 ± 0.1, 9.5 ± 0.1 and 10.5 ± 0.1) was measured to be 2.7 ± 0.02 μm, 1.8 ± 0.02 μm, and 1.4 ± 0.02 μm, respectively. As the pH value increased, the crystallite size (D) of the prepared CuO thin films also increased, ranging from 19.25 nm to 25.63 nm. As the pH value increased from 8.5 ± 0.1 to 10.5 ± 0.1, the bandgap (E_g_) value decreased from 1.52 eV to 1.42 eV. The film deposited at a pH of 10.5 ± 0.1 exhibited a higher refractive index (n = 3.2) and extinction coefficient (K = 0.82) compared to those deposited at pH 8.5 ± 0.1 (n = 2.8; K = 0.76) and pH 9.5 ± 0.1 (n = 2.9; K = 0.77). The CuO photocathode deposited at pH 10.5 ± 0.1 shows maximum photocurrent density of 1.45 mA/cm^2^ at −0.1 V vs. RHE in 0.5 M Na_2_SO_4_ solution. The CuO deposited at pH 10.5 ± 0.1 photocurrent degradation was reduced from 70 % compared pH 8.5 ± 0.1 (20 %) and pH 9.5 ± 0.1 (50 %) as observed from the chronoamperometric curve. Furthermore, the EIS analysis shows, the CuO (pH 10.5 ± 0.1) electrode material has high conductivity value of 0.6862 S/cm compared CuO at pH 8.5 ± 0.1 (0.2779 S/cm) and CuO at pH 9.5 ± 0.1 (0.4646 S/cm) electrodes.

## Data availability

The datasets generated during and/or analysed during the current study are available.

## CRediT authorship contribution statement

**E. Arulkumar:** Writing – original draft, Software, Methodology, Formal analysis, Data curation, Conceptualization. **S. Thanikaikarasan:** Writing – review & editing, Validation, Supervision, Resources, Investigation, Data curation, Conceptualization. **S. Rajkumar:** Validation, Investigation, Data curation. **Wasihun Wondimu:** Software, Investigation, Data curation.

## Declaration of competing interest

The authors declare the following financial interests/personal relationships which may be considered as potential competing interests:**Dr.Sethuramachandran Thanikaikarasan** reports **equipment**, **drugs**, or **supplies** was provided by **Saveetha School of Engineering. Dr.S.Rajkumar** reports a relationship with **Hawassa University** that includes: employment and non-financial support. **Dr.Sethuramachandran Thanikaikarasan** has **patent No** pending to **Nil**. **Dr.S.Rajkumar** (**Co-author**) not employed in any **pharma company.** If there are other authors, they declare that they have no known competing financial interests or personal relationships that could have appeared to influence the work reported in this paper.
